# Pre-Sowing Treatment of *Tilia cordata* Mill. by Seed Material Fermentation

**DOI:** 10.3390/plants11212880

**Published:** 2022-10-28

**Authors:** František Bednařík, Kateřina Houšková, Lena Bezděčková, Oldřich Mauer

**Affiliations:** 1 Department of Silviculture, Faculty of Forestry and Wood Technology, Mendel University in Brno, Zemědělská 1, 613 00 Brno, Czech Republic; 2 Research Institute Cannasan, z.ú., Třída Svobody 737, Malenovice, 736 02 Zlín, Czech Republic; 3 Department of Food Analysis and Chemistry, Faculty of Technology, Tomas Bata University in Zlín, Vavrečkova 5669, 760 01 Zlín, Czech Republic; 4 Research Station Kunovice, Forestry and Game Management Research Institute, v.v.i. Strnady, Na Záhonech 601, 686 04 Kunovice, Czech Republic

**Keywords:** abscisic acid, germination, maturity, seed, small-leaved lime

## Abstract

The aim of the work was to verify whether the fermentation of seed material is a suitable pre-sowing treatment of small-leaved lime. Seed material of two origins was treated for a period of 7, 14 and 21 days using anaerobic fermentation and for 10, 20 and 30 days using aerobic fermentation, the control treatment being the seed after warm–cold stratification. The water content, germination capacity and abscisic acid level of the seed were evaluated, together with the emergence and morphological parameters of annual lime seedlings that had emerged from the tested seeds. It was found that fermented seed had a low abscisic acid level (up to 700 pmol/g), a higher germination capacity (approx. 90%) than mature seed without fermentation and the morphological parameters of the seedlings that had emerged from seed after fermentation reached higher values, compared to those of the seedlings that had emerged from seed after warm–cold stratification. The seed material had to be collected green (e.g., immature seeds) and moistened. A suitable anaerobic fermentation time is 7 days; the aerobic phase can last 10, 20 or 30 days. The fermentation of seed material can shorten the preparation of seed for sowing by 1 year.

## 1. Introduction

The seeds of some broadleaves are dormant and therefore require pre-sowing preparation. The seeds of the small-leaved lime (*Tilia cordata* Mill.) are also dormant and do not germinate after the fall, i.e., they do not germinate at the beginning of the growing season following the year of maturation, but instead, the next. Heit [1] states that they can maintain a viability for more than 5 years on the soil surface. The lime seed dormancy is caused by the hard pericarp and the impermeable seed coat (physical dormancy), and the dormant embryo (physiological dormancy), which is brought about by the high content of abscisic acid (ABA) in the embryo [2,3]. According to Nikolaeva et al. [4], seeds of *Tilia cordata* have either physiological and physical dormancy, or only physiological dormancy. In any case, seed for planting stock cultivation (i.e., for artificial regeneration) requires a long pre-sowing treatment.

Vincent [5] states that it is necessary to collect the seed of small-leaved lime in mid-October, then to soak it, stratify it for a period of 2 months at 15–25 °C, and then for 5 months at 4–8 °C until spring sowing. Nevertheless, even at this very early collection time (the seed had a water content of about 20%), its dormancy could not be completely overcome, and the germination capacity was between 25% and 75%. According to this author, the lime dormancy cannot be overcome by removing the pericarp and subsequent stratification in moist sand, as recommended by Spaeth [6], for the seed of the American lime (*Tilia americana* L.). Vanstone [7] recommends stratification of the small-leaved lime in a mixture of peat and sand with a ratio of 1:1 (by volume) and a moisture of 30% (by weight). Walter [8] mentions soaking fully mature lime seeds for 24 h in October, and then stratifying them at 4–6 °C until spring sowing. According to Flemer [9], the pre-sowing treatment of lime is successful if the seed is closed in a wooden container with moist sand after collection and sown the following autumn.

However, most authors describe warm–cold stratification without a medium as the most appropriate way to overcome small-leaved lime seed dormancy. This pre-sowing treatment takes 8 to 9 months [3]. To reduce the effects of impermeable pericarp, Schopmeyer [10] suggests soaking fully mature seed in water for 48 h and then treating it with warm stratification at 20–25 °C for 60 days. Dirr and Heuser [11] consider the best time of warm stratification to be 3–5 months, and Suszka et al. [3] mention warm stratification of hydrated seed at 20–25 °C for 4 months. According to Tylkowski [12], warm stratification can be substituted by maceration (chemical scarification) in concentrated sulfuric acid. Heit [13] recommends macerating seed for 10–50 min at 23–27 °C, where lower temperatures require longer maceration. According to this author, the time of maceration depends on the degree of impermeability of the seed coat, which is influenced, for example, by the seed origin, the collection time and the storage conditions, including temperature and humidity. The degree of impermeability of the seed coat and the time of maceration can be determined according to the rate of seed water saturation within 1–2 days. Heit [13] does not recommend mechanical scarification and soaking of the seed in hot water because it is not as effective as maceration. Nevertheless, Shepperd [14] suggests that lime seed be scarified and sown immediately after collection (in the autumn) or stratified before the spring sowing. In either case, warm stratification should also be followed by a cold phase at 4 °C [15] for 3 months [11]. Suszka et al. [3] report a temperature of 3 °C for 14–18 weeks and pre-sowing treatment should end upon the start of the germination of the first seeds. Shepperd [14] admits a lower temperature of 1–3 °C for about 3 months.

The seed of the small-leaved lime is usually collected when fully mature. Schubert [16] recommends that it be collected according to the actual state of maturity of the embryo—the yellowish embryo should fill the entire cavity of the white endosperm. According to this author, leaving the fruit on the tree longer reduces its moisture, thus deepening the germination barriers. However, lime seed, as well as ash and hornbeam, can be collected “green” and sown immature, when they are already fully developed but not fully mature [3]. The aim of the early harvest is to collect the seed before germination inhibitors accumulate inside. The immature seeds have semi-permeable seed coats allowing water and oxygen to penetrate the embryo, the embryo is fully developed and simple organic substances—carbohydrates and amino acids—are found in the endosperm [17]. According to Šebánek [17], abscisic acid levels rise in maturing seeds due to their desiccation. ABA inhibits alpha-amylase synthesis in mature seeds and induces the synthesis of proteins and other polymeric reserve substances—polysaccharides and lipids [17]. As moisture decreases, physiological dormancy gradually occurs [18] and seed coats are cutinized. The seed coats gradually become impermeable and physical dormancy occurs.

The conditions for the success of immature seed material collection are that the water content of the seed must not decrease distinctly (i.e., it must remain high) and the seed must not become moldy or be exposed to sudden temperature changes [19]. According to this author, the seed must be sown immediately after collection, no additional parts such as bracts or wings are removed; they are not suitable for storage because they quickly lose their germination capacity. Lime nuts (fruit) are collected and sown from the last week of August until the end of the first third of September, when brown spots begin to appear on them; at this stage of maturity, their germination capacity is at about 70% [19]. In addition, until the spring months, sowing must be protected from damage by insects, rodents and from over-wetting due to autumn, winter and early spring rainfall. Seedling emergence is strongly influenced by weather conditions. If seeds are unevenly sown, those near the soil surface can dry out or freeze, while those in deeper layers may die due to lack of oxygen especially if waterlogged [19].

The seeds of some tree species pass through the digestive tract of birds in order to germinate successfully. Birds consume fruit (including seeds) and secrete highly germinating seeds after digesting the pericarp [20,21]. During the digestion, the seeds remain in the intestinal tract for some time, i.e., in an environment where intensive anaerobic fermentation takes place at a temperature of about 37–39 °C, i.e., the basal temperature of birds [21]. Therefore, the use of seeds collected immaturely, in combination with subsequent controlled fermentation, appears to overcome the complex of physical and (morpho)physiological dormancy and shorten the time of pre-sowing seed treatment. The aim of the work was to verify whether the fermentation of immature seed material is a suitable method for pre-sowing treatment of small-leaved lime, however, the possible effect of chemical substances (e.g., digestive enzymes found in the digestive tract of birds) was not investigated.

## 2. Results

During maturation, the water content in the seeds decreased (Table 1). In the immature state, when the seed material was collected for fermentation, the water content in the seeds was 55–59%; when mature seeds were collected for warm–cold stratification, the water content was 12–15%. At the time of germination after seed moistening, the seed water content was 78%, 82% and 81% in Experiment 1, 2 and 3, respectively (the results are not presented in tables).

All pre-sowing treatments, with fermentation and without moistening of the seed material, were repeatedly unsuccessful. The seeds died during the fermentation process (confirmed by the cut test) and therefore their germination capacity was zero (Table 2). Seeds died even when the anaerobic fermentation lasted 14 days or longer (0% germination capacity). However, pre-sowing treatment of seeds from both sources was always successful if the seed material was moistened; anaerobic fermentation lasted 7 days and was followed by the aerobic fermentation for 10, 20 or 30 days (prospective treatments A–F in Appendix A in Table A1); the seed germination capacity was statistically significantly the highest, i.e., about 90%. No statistically significant difference was found between these prospective treatments with different durations of the aerobic fermentation and seed origins, *p* > 0.05. The seed germination capacity after warm–cold stratification was lower than in all treatments after successful fermentation (*p* = 0.000); it was about 68% in Experiment 1 and about 80% in Experiments 2 and 3.

The level of abscisic acid, after the collection of lime seed material at the state of immaturity, reached 1379 pmol/g (Table 3). It decreased after moistening, even more after 7 days of anaerobic fermentation, followed by a 10-, 20- or 30-day aerobic phase. A further reduction then occurred during seed storage at 0–2 °C (always <200 pmol/g after 4.5 months). After collection of seed material at full maturity, the level of abscisic acid was the highest (7201 pmol/g).

**Figure 1:**
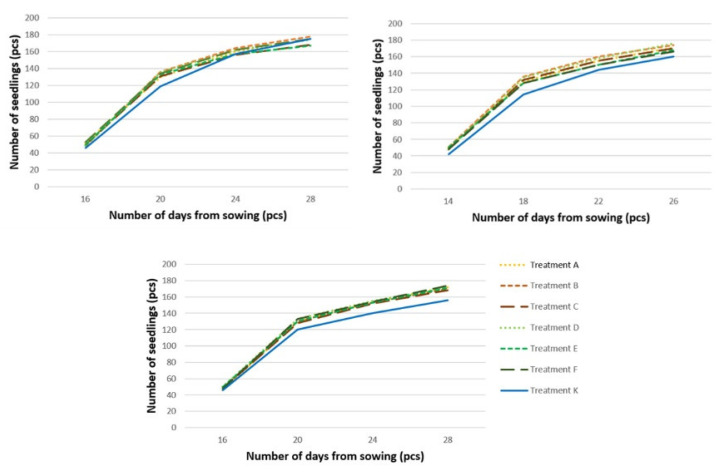
Emergence of seedlings grown from seeds after different pre-sowing treatments: a moistening, 7-day anaerobic and 10-day aerobic fermentation of seeds from Uherské Hradiště, B moistening, 7-day anaerobic and 20-day aerobic fermentation of seeds from Uherské Hradiště, C moistening, 7-day anaerobic and 30-day aerobic fermentation of seeds from Uherské Hradiště, D moistening, 7-day anaerobic and 10-day aerobic fermentation of seeds from Slušovice, E moistening, 7-day anaerobic and 20-day aerobic fermentation of seeds from Slušovice, F moistening, 7-day anaerobic and 30-day aerobic fermentation of seeds from Slušovice, K warm-cold stratification of seeds from Uherské Hradiště.

The total number of seedlings that had emerged from seed after pre-sowing treatment by fermentation (prospective treatments A–F in the Appendix A Table A1) was comparable to the number of seedlings that had emerged from seeds after classic warm–cold stratification (treatment K) in Experiment 1, or slightly higher in Experiments 2 and 3 with a difference of up to 10% (Figure 1). The emergence was 80–85%. Seedlings always grew slightly slower after warm–cold stratification of seed.

**Figure 2:**
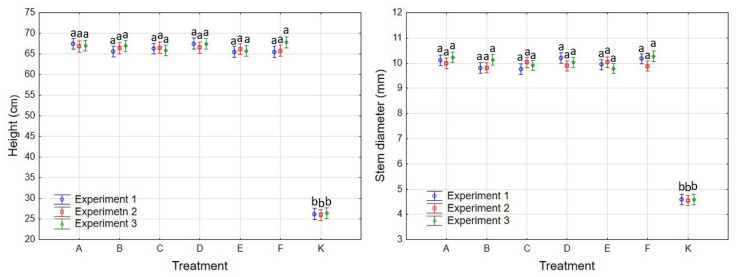
The height (**left**) and the stem diameter (**right**) of seedlings that had emerged from seed after different pre-sowing treatments: A moistening, 7-day anaerobic and 10-day aerobic fermentation of seeds from Uherské Hradiště, B moistening, 7-day anaerobic and 20-day aerobic fermentation of seeds from Uherské Hradiště, C moistening, 7-day anaerobic and 30-day aerobic fermentation of seeds from Uherské Hradiště, D moistening, 7-day anaerobic and 10-day aerobic fermentation of seeds from Slušovice, E moistening, 7-day anaerobic and 20-day aerobic fermentation of seeds from Slušovice, F moistening, 7-day anaerobic and 30-day aerobic fermentation of seeds from Slušovice, K warm-cold stratification of seeds from Uherské Hradiště at the end of the growing season in Experiments 1, 2 and 3 (different letter indexes denote statistically significant differences between the various treatments).

In Experiments 1 (top left), 2 (top right) and 3 (bottom). The height of the seedlings that had emerged from seed after pre-sowing treatment by fermentation (treatments A–F) was always comparable in all treatments at the end of the first vegetation period after sowing (*p* > 0.05), reaching values of about 67 cm (Figure 2). The seedlings that had emerged from seeds after classical warm–cold stratification (treatment K) were always statistically significantly smaller (*p* = 0.00), reaching a height of about 27 cm.

The seedling stem diameter that had emerged from seed, prepared by the fermentation of seed material (A–F), was always around 10 mm after the first growing season; there were no statistically significant differences (*p* > 0.05) between the treatments (Figure 2). The stem diameter of the seedlings that had emerged from seed after classical warm–cold stratification was statistically significantly lower (*p* = 0.00), reaching a value of about 4.5 mm.

## 3. Discussion

The increasing proportion of broadleaves and the biodiversity of forests require more efficient processing of seed material and the pre-sowing treatment of some tree species. Small-leaved lime seeds at full maturity show deep dormancy due to impermeable pericarp and seed coat and high abscisic acid (growth inhibitor) content in the embryo [2,3]. For this reason, the seed requires a long warm–cold stratification and is not ready for sowing until the second spring following collection. Long pre-sowing treatment, or deep dormancy, can be avoided by collecting immature seeds and sowing them immediately after collection. However, the sowing of immature seeds is associated with greater seed mortality and is not always successful, as seeds are very sensitive to temperature, humidity and a lack of air at the time of collection [22]. The solution seems to be the fermentation of the seed material when the seeds are immature.

To obtain high-quality lime seed, it is necessary to determine the correct collection time. In the course of maturation, the water content of the seeds on the trees naturally decreases [23,24]; in the case of the seed tested, it decreased down to 12–15% at the time of full maturity. However, if the seed is collected earlier (i.e., immature) the water content is higher. Our analyses show that, at this stage of maturity, when the pericarp is green and the seeds are brown, lime seed has a water content of 55–60%. The best germination capacity was also achieved by seeds of American lime (*Tilia americana* L.) as soon as the seed coats turned from green to completely brown, but the seed had not yet dropped from the trees [25]. The germination capacity was very low, or zero, if the seed had matured to the point where it dropped, or could be shaken, from the tree.

The high water content in the immature seeds (55–60%) should enable the seed to overcome dormancy. Jensen [26] recommends a water content of 40–43% for stratification of fully matured small-leaved lime seeds. However, the dormancy of the immature seeds seems to be weak (low ABA content, apparently weaker pericarp and seed coat) and the fermentation process seems to ensure particularly suitable conditions for the maturation of the embryo without deepening the dormancy. Embryonal (or internal) dormancy is caused not only by inhibitors within the embryo or surrounding tissue, but also by an immaturity of the embryo [23], i.e., morphophysiological dormancy. Even though the water content in the seed used in our experiment was high (55–60%) at the time of collection of immature seeds, it was insufficient for successful fermentation of the seed material. Fermentation, or rather its anaerobic stage (temperature 37–39 °C without access to air), without the moistening of the seed material, caused the death of the seed, and therefore the material had to be soaked in water for 24 h beforehand.

The results suggest that, in immature lime seed material, it is possible to overcome its dormancy very quickly (within approx. 30 days) by two-phase controlled fermentation after moistening the seed material. The seeds remained alive during the 7-day anaerobic stage of the fermentation process at 37–39 °C, which is contrary to previous experience with seeds harvested at this stage of maturity [8]. During the pre-sowing treatment by fermentation, the development of the embryo is apparently completed and the ABA level in the seeds decreases at the same time. A decrease in the level of ABA usually occurs during cold stratification at low temperatures, i.e., 3–5 °C [3,27]. However, e.g., Shun-Ying Chen et al. [28] confirmed the reduction of ABA also during the warm phase of stratification. However, the level of ABA was not already high at the time of the collection of immature seed in our experiment; compared to the level at full maturity, it was 5 times lower. However, the absolute value of the ABA content is not a decisive indicator of seed dormancy and the capacity to germinate [27,29]. Rodriguez-Gacio et al. [30] consider a balance between ABA and gibberellins as crucial. ABA promotes the induction of dormancy and suppresses germination, while gibberellins promote the breaking of dormancy and the capacity to germinate [31]. However, it seems that the difference between the level of ABA, when the seeds are still immature, and its further decrease during the fermentation in the framework of this research (up to 700 pmol/g), and the level of ABA at the state of full maturity (approx. 7200 pmol/g) is clear evidence of the fact that the seeds are non-dormant. Shun-Ying Chen et al. [28], however, found that the total ABA content of red bayberry (*Myrica rubra* (Lour.) Siebold & Zucc.) seeds subjected to warm or cold stratification, or both, was 8.7 to 14.0 times lower than that of fresh seed.

The results also clearly showed that the seeds remained alive only after the shortest time of anaerobic fermentation, i.e., after 7 days. It appears that the time of anaerobic fermentation could be even shorter than 7 days, since the time during which the seeds of endozoochorous plants pass through the digestive tract of birds is 3–4 days according to Miller-Schneider [21], or, according to Opravil and Drchal [32], even just a few minutes or hours. However, Varela and Bucher [33] point out that the conditions in the digestive tract make it possible to overcome seed coat dormancy and, in some cases, the passage time through the tract may be insufficient. Barnea et al. [34] also claim that a longer stay in the digestive tract can have a positive effect on seed germination, which is apparently related to a more efficient disruption of the seed coats. Moreover, the outcome of seed passage through the digestive tract may depend on the animal species that consume it [35]. The dental formula of herbivorous mammals is modified in several ways: to crush, grind or shred plant matter, so the chemical environment of the digestive tract is also modified to extract nutrients in an effective way, usually harboring symbiotic bacteria and protozoa, which can digest the structural polymers of the cell wall through fermentation [36]. Kleyheeg et al. [37] also confirmed the positive effect of the digestive tract environment on the seed coat dormancy, but warn that, after overcoming it, the seeds in the digestive tract can then be negatively influenced (i.e., damaged) chemically. This could also explain our result that in both longer treatments of 14 and 21 days of anaerobic fermentation, the seeds were dead.

The duration (10, 20, 30 days) of the second stage of the fermentation process controlled by our team—aerobic fermentation—had no effect on the resulting seed germination and the emergence of seedlings. The result was high seed germination capacity (approx. 90%), higher than after warm-cold stratification. The fermentation process therefore appears to be a more effective pre-sowing treatment. Although the emergence of seedlings from pre-germinated seeds was not much affected by pre-sowing treatment, seedlings emerged slightly faster again after seed fermentation, but one-year-old seedlings grown from fermented seeds reached a significantly greater height and stem diameter. The fermentation process obviously affected the physiological quality of the seeds, but the essence of this change was not investigated. With regard to the innovativeness of the pre-sowing treatment of lime with a fermentation process, a patent was obtained for the described technology [38], and it appears that the fermentation of seed material could also be a solution for the seeds of other tree species with problematic dormancy and a long and complicated pre-sowing treatment (elms, European hornbeam, European ash, etc.).

## 4. Materials and Methods

### 4.1. Materials

Two small-leaved lime trees, about 60 km apart, were selected as the source of seeds for this experiment. The first tree, which was about 50 to 55 years old, was located on the SW edge of one stand (at an altitude of 180 m a.s.l.) in the natural forest area 36 “Central Moravian Carpathians” in the cadastral area of Uherské Hradiště, Czech Republic (N 49°05′24″, E 17°26′59″, with the origin marked “Uherské Hradiště”). The second tree, which was about 45 to 50 years old, was located on the south edge of one stand (at an altitude of 330 m a.s.l.) in the natural forest area 41 “Hostýn-Vsetín highlands and Javorníky”, in the cadastral area of Slušovice, Czech Republic (N 49°15′36″, E 17°48′38″, with the origin marked “Slušovice”).

### 4.2. Methods

**Figure 3:**
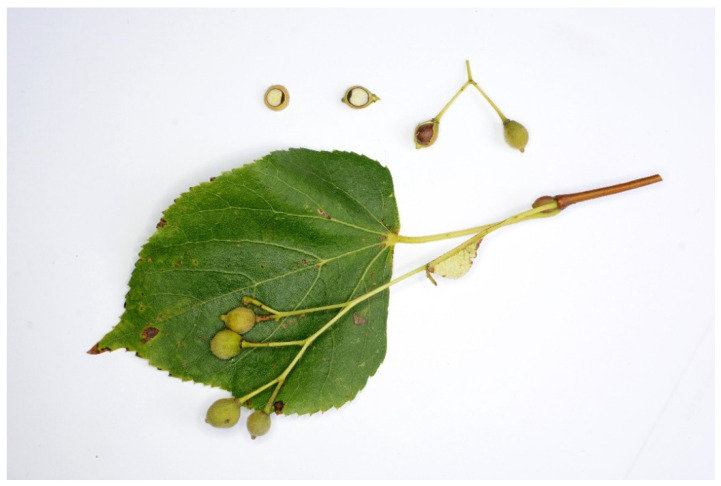
Seed material of small-leaved lime with the right maturity, suitable for fermentation.

Seed material from both, i.e., a mixture of fruits, bracts and leaves, was collected directly from mother trees when seeds were still immature. The right maturity of the seeds for fermentation was characterized by the following features: green color of the fruit, brown color of the seed coat, milk does not flow out after squeezing between the fingers, internal tissues of seeds are soft, the developed embryo is clearly visible in the cross section of the seed, the seed coats are not yet fully cutinized (Figure 3). This state of seed immaturity was seen at both sites at the turn of August and September. Mature seeds were collected the previous year at the turn of November and December; they served as a control treatment (Table 4). The full maturity was characterized by the following features: brown color of the fruit, brown color of the seed coat, internal tissues of seeds are solid, the developed embryo is clearly visible in the cross section of the seed, the seed coats are fully cutinized.

Two hours after collection, half of the immature seed material was moistened by immersing it in water at 15 °C for 24 h and then left on a sieve for 1 h to remove excess water. The second half of the seed material was not moistened with water after collection. Both moistened and non-moistened seed material underwent a fermentation process that was monitored and regulated by the STC15Re control unit with the PSM-01 psychrometer (ELBEZ, s.r.o. company, Czech Republic, 2005) and consisted of two basic phases:

1. Anaerobic fermentation at a temperature of 37–39 °C. During this phase, the seed material was put inside in an airtight plastic container and placed in a thermobox at 37–39 °C for 7, 14 and 21 days. The relative air humidity inside reached 95–100%. After the end of the anaerobic fermentation, the seed material from the individual experimental treatment was taken out of the plastic containers and underwent aerobic fermentation;
2. Aerobic fermentation at a temperature of 20–25 °C. The seed material was placed in a thermobox with active ventilation. The temperature inside was 20–25 °C, the relative air humidity was 90–95% and the duration of the aerobic fermentation was 10, 20 and 30 days.

At the end of the entire fermentation process, the seeds were separated from the other material on a shaker and stored without any medium in a cooling box at a temperature of 0–2 °C until the spring sowing. Fully mature seed, collected at the stage of full maturity in the year before the collection of immature seeds (at the turn of November and December), from the same source (tree) as the seed originating from Uherské Hradiště, served as a control treatment. This seed was prepared by classical warm–cold stratification (130 days at 20–25 °C, 160 days at 2–4 °C).

A total of 36 pre-sowing treatments by fermentation of seed material were tested, depending on the seed source (i.e., of Uherské Hradiště and Slušovice origins), the time of anaerobic fermentation (7, 14 and 21 days) and the time of aerobic fermentation (10, 20 and 30 days), whether or not the seed material was moistened after collection (Table A1 in Appendix). For each treatment, 3 kg of seed material was collected and divided further into 3 replicates of 1 kg each. The total amount of seed material collected was 108 kg (54 kg from each source of seeds, i.e., tree. The entire experiment was repeated in 3 consecutive years, the results being marked as Experiments 1, 2 and 3 (Table 4).

During seed maturation, from the immature state to full maturity, and at the time of germination before the spring sowing, two seed samples weighing 5 g each were taken, in accordance with ISTA [39], in order to determine their water content. Water content means the proportion of the weight of the water in the working sample of the seeds, expressed in % of the original fresh weight of the working sample.

After the pre-sowing treatments, the germination capacity of seeds was determined using a germination test in which 4 × 100 seeds were placed in plastic germination boxes, in accordance with ISTA [39]. The temperature, light conditions and medium during the germination test differed from those in the germination test stated in ISTA [39], i.e., the temperature was stable at +12 °C (Sanyo Incubator Mir-153), with light and dark alternating every 12 h and the seeds in the germination boxes (17 × 17 × 3 cm) were placed on two layers of moistened filter paper. Germination capacity was calculated as the number of germinated seeds at the end of the germination test (after 21 days) out of the total number of seeds (in %). A seed that had a radicle longer than the length of the seed was considered germinated. Seeds that did not germinate until the end of the germination test underwent a cut test. Cutting of ungerminated seeds was performed to ensure that the internal contents of the seeds were rotting (liquid, dark) and the seeds were dead.

During the pre-sowing treatment and storage of the seeds originating from Uherské Hradiště, in Experiment 2 of the selected prospective treatments (A, B and C in Table A1 in Appendix A), i.e., of those whose germination capacity was not zero, seed samples were taken in order to determine the ABA content. Eleven samples were taken from the seed before, during and after fermentation: 1. at the time of its harvest (10 September); 2. after additional moistening of the seed material (11 September); 3.–5. after the end of the 7-day anaerobic and 10-, 20- and 30-day aerobic fermentation; 6.–11. after 2, 4 and 4.5 months of storage at 0–2 °C in treatments with 10 and 30 days of aerobic fermentation. Samples were also taken from seeds at the beginning of full maturity (30 October). The sampling procedure first consisted of taking 6 primary samples from each treatment after thorough mixing of the seed. The seed samples taken for the ABA content analysis were marked and wrapped in aluminum foil and then stored in a Dewar Vessel with liquid nitrogen. In the laboratory, 6 samples were mixed together and 2 g of this mixed sample was taken as a working sample for the determination of the ABA content, which was analysed at the Institute of Experimental Botany of the Czech Academy of Sciences using the LC–MS method described by Vondráková et al. [27].

In the middle of March, the year following the collection of immature seeds, 200 seeds of all prospective fermented treatments (A–F in Table A1 in the Appendix A) were sown in a bed (N 49°16′09″, E 17°48′08″) on the surface of a 35 cm layer of a highly organic substrate consisting of 20% peat, 20% composted manure and 60% loamy mineral soil and they were covered with the same substrate, with the thickness of the layer corresponding to twice the length of the seeds. The control treatment was the sowing of seeds collected at full maturity (K in Table A1 in the Appendix A) and prepared by warm–cold stratification, which lasted 290 days (as described above) and was started at the beginning of July in the year following their harvest (Table 4). The sowing of stratified and fermented seeds was thus carried out at the same time and after pre-germination of the seeds at 12 °C with continuous moistening. Only seeds with a clearly developing sprout were sown. After sowing, the emergence of the seedlings was monitored, i.e., the number of plants that had emerged within about 14 days after sowing and every 4 days until about 1 month after sowing. At the end of the growing season (in October—the increment of seedlings without leaves was finished), the height of the above-ground part (i.e., the length from the root collar to the top of the terminal bud in cm) and the stem diameter 1 cm above the root Seed water content, germination capacity, emergence and seedling parameters were tested for 3 consecutive years (Experiments 1, 2 and 3). The content of ABA was determined only in Experiment 2. Data for the ABA content, germination capacity and seedling emergence were processed in MS Excel [40]. To compare the germination capacity of the prospective and control treatments, the data were transformed to normal distribution using the arcsine transformation and further compared using two-factor ANOVA (testing year–1st factor; pre-sowing treatment–2nd factor), significant differences between individual treatments were found using Tukey’s HSD test in Statistica 12.0 [41]. The plant heights and stem diameters of the individual treatments were tested using two-factor ANOVA (testing year–1st factor; pre-sowing treatment–2nd factor) and Tukey’s test without any data transformation, after meeting the common data assumptions. The same significance level (α) of 0.05 was chosen in all statistical tests. Vertical columns in the statistical diagrams represent 95% confidence intervals.

## 5. Conclusions

Fermentation of small-leaved lime seed material collected immature, consisting of moistening, 7-day of anaerobic and 10–30-day of aerobic phases, appears to be effective pre-sowing treatment, more advantageous than the classic warm-cold stratification of fully matured seed, or the sowing of seed material collected immature without any pre-sowing treatment. The seeds are non-dormant after fermentation and are thus ready for sowing in the spring following collection, which shortens the time for seed preparation for sowing by 1 year. In addition, one-year-old seedlings from fermented seeds achieve better morphological parameters, which makes it possible to obtain planting material that is suitable for planting after 1 year of cultivation. It is assumed that the fermentation of seed material could significantly increase the efficiency of the pre-sowing treatment of also other tree species with dormant seeds.

## Figures and Tables

**Table 1:** Water content (%) of seeds during the maturation, on the trees of both origins, in different years of the research.

Year	Seed Origin	Seed Water Content (%)			
**2010**		**29 August ^1^**	**12 September**	**26 September**	**5 December ^2^**
	**Uherské Hradiště**	57%	47%	35%	15%
	**Slušovice**	59%	48%	37%	-
**2011**		**10 September ^1^**	**24 September**	**30 October**	**28 November ^2^**
	**Uherské Hradiště**	57%	49%	35%	12%
	**Slušovice**	58%	50%	35%	-
**2012**		**5 September ^1^**	**20 September**	**25 October**	**30 November ^2^**
	**Uherské Hradiště**	55%	46%	33%	15%
	**Slušovice**	57%	47%	34%	-

^1^ time of seed material collection in immature state. ^2^ time of seed material collection in mature state.

**Table 2:** Seed germination capacity in tested pre-sowing treatments in Experiment 1, 2 and 3 (different statistical letter indexes denote statistical differences between the various treatments in each graph).

Source of Seed (Origin)	Moistening of Seed Material after Collection	Fermentation of Seed Material		Germination Capacity (%)		
		Duration of the Anaerobic Phase	Duration of the Aerobic Phase	Experiment 1	Experiment 2	Experiment 3
Uherské Hradiště	Yes	7 days	10 days	89.3 ^a^	89.8 ^a^	90.5 ^a^
			20 days	88.8 ^a^	90.3 ^a^	90.3 ^a^
			30 days	89.5 ^a^	90.0 ^a^	90.5 ^a^
		14 days	10 days	0.0 ^d^	0.0 ^d^	0.0 ^d^
			20 days	0.0 ^d^	0.0 ^d^	0.0 ^d^
			30 days	0.0 ^d^	0.0 ^d^	0.0 ^d^
		21 days	10 days	0.0 ^d^	0.0 ^d^	0.0 ^d^
			20 days	0.0 ^d^	0.0 ^d^	0.0 ^d^
			30 days	0.0 ^d^	0.0 ^d^	0.0 ^d^
	No	7 days	10 days	0.0 ^d^	0.0 ^d^	0.0 ^d^
			20 days	0.0 ^d^	0.0 ^d^	0.0 ^d^
			30 days	0.0 ^d^	0.0 ^d^	0.0 ^d^
		14 days	10 days	0.0 ^d^	0.0 ^d^	0.0 ^d^
			20 days	0.0 ^d^	0.0 ^d^	0.0 ^d^
			30 days	0.0 ^d^	0.0 ^d^	0.0 ^d^
		21 days	10 days	0.0 ^d^	0.0 ^d^	0.0 ^d^
			20 days	0.0 ^d^	0.0 ^d^	0.0 ^d^
			30 days	0.0 ^d^	0.0 ^d^	0.0 ^d^
Slušovice	Yes	7 days	10 days	0.0 ^d^	0.0 ^d^	0.0 ^d^
			20 days	0.0 ^d^	0.0 ^d^	0.0 ^d^
			30 days	0.0 ^d^	0.0 ^d^	0.0 ^d^
		14 days	10 days	0.0 ^d^	0.0 ^d^	0.0 ^d^
			20 days	0.0 ^d^	0.0 ^d^	0.0 ^d^
			30 days	0.0 ^d^	0.0 ^d^	0.0 ^d^
		21 days	10 days	0.0 ^d^	0.0 ^d^	0.0 ^d^
			20 days	0.0 ^d^	0.0 ^d^	0.0 ^d^
			30 days	0.0 ^d^	0.0 ^d^	0.0 ^d^
	No	7 days	10 days	0.0 ^d^	0.0 ^d^	0.0 ^d^
			20 days	0.0 ^d^	0.0 ^d^	0.0 ^d^
			30 days	0.0 ^d^	0.0 ^d^	0.0 ^d^
		14 days	10 days	0.0 ^d^	0.0 ^d^	0.0 ^d^
			20 days	0.0 ^d^	0.0 ^d^	0.0 ^d^
			30 days	0.0 ^d^	0.0 ^d^	0.0 ^d^
		21 days	10 days	0.0 ^d^	0.0 ^d^	0.0 ^d^
			20 days	0.0 ^d^	0.0 ^d^	0.0 ^d^
			30 days	0.0 ^d^	0.0 ^d^	0.0 ^d^
		**Warm–cold stratification of seed**				
		**Duration of the warm phase**	**Duration of the cold phase**			
Uherské Hradiště	No	130 days	160 days	67.3 ^c^	80.3 ^b^	80.8 ^b^

**Table 3:** Content of abscisic acid in the seed of Uherské Hradiště origin after its collection in the state of immaturity, after its moistening and fermentation, during storage and after collection in full maturity (Experiment 2).

State of Seed Maturity, Pre-Sowing Treatment	Seed Storage Duration	ABA Content (pmol/g)
Immature seeds without any pre-sowing treatment	0 months	1379
Immature seeds after moistening	0 months	1244
Immature seeds after moistening, 7-day anaerobic and 10-day aerobic fermentation	0 months	689
	2 months	439
	4 months	340
	4.5 months	178
Immature seeds after moistening, 7-day anaerobic and 20-day aerobic fermentation	0 months	149
Immature seeds after moistening, 7-day anaerobic and 3-day aerobic fermentation	0 months	272
	2 months	246
	4 months	234
	4.5 months	121
Mature seeds without any pre-sowing treatment	0 months	7201

**Table 4:** A time overview of the main research field work in Experiment 1, 2 and 3.

	Experiment 1	Experiment 2	Experiment 3
	Treatments with Fermentation		
Seed material collection	29 August 2010	10 September 2011	5 September 2012
Beginning of the pre-sowing treatment	29 August 2010	10 September 2011	5 September 2012
Seed sowing	March 2011	March 2012	March 2013
Seedling measurement	October 2011	October 2012	October 2013
	**Treatments with warm–cold stratification**		
Seed material collection	November 2009	November 2010	December 2011
Beginning of the pre-sowing treatment	July 2010	July 2011	July 2012
Seed sowing	March 2011	March 2012	March 2013
Seedling measurement	October 2011	October 2012	October 2013

## Data Availability

Data used in this article are available from the authors.

## Appendix A

**Table A1:** An overview of tested pre-sowing treatments.

Source of Seed (Origin)	Moistening of Seed Material after Collection	Fermentation of Seed Material		Designation of Prospective Treatments *
		Duration of the Anaerobic Phase	Duration of the Aerobic Phase	
Uherské Hradiště	Yes	7 days	10 days	A
			20 days	B
			30 days	C
		14 days	10 days	
			20 days	
			30 days	
		21 days	10 days	
			20 days	
			30 days	
	No	7 days	10 days	
			20 days	
			30 days	
		14 days	10 days	
			20 days	
			30 days	
		21 days	10 days	
			20 days	
			30 days	
Slušovice	Yes	7 days	10 days	D
			20 days	E
			30 days	F
		14 days	10 days	
			20 days	
			30 days	
		21 days	10 days	
			20 days	
			30 days	
	No	7 days	10 days	
			20 days	
			30 days	
		14 days	10 days	
			20 days	
			30 days	
		21 days	10 days	
			20 days	
			30 days	
		**Warm-cold stratification of seed**		
		**Duration of the warm phase**	**Duration of the cold phase**	
Uherské Hradiště	No	130 days	160 days	K

* Prospective pre-sowing treatments whose seeds were germinable and tested in terms of emergence and growth of seedlings after sowing.
